# Crop and non-crop productivity in a traditional maize agroecosystem of the highland of Mexico

**DOI:** 10.1186/1746-4269-5-38

**Published:** 2009-11-27

**Authors:** Rosa M González-Amaro, Angélica Martínez-Bernal, Francisco Basurto-Peña, Heike Vibrans

**Affiliations:** 1 Postgrado en Botánica, Colegio de Postgraduados en Ciencias Agrícolas, 56230 Montecillo, Estado de México, México; 2 Departamento de Biología, División de Ciencias Biológicas y de la Salud, Universidad Autónoma Metropolitana-Iztapalapa, Apartado Postal 55-535, 09340, DF, México; 3 Jardín Botánico, Instituto de Biología, Universidad Nacional Autónoma de México, Apartado Postal 70-614, DF, México

## Abstract

**Background:**

In Mexico, the traditional maize cultivation system has resisted intensification attempts for many decades in some areas, even in some well-connected regions of the temperate highlands. We suggest that this is due to economics.

**Methods:**

The total useful biomass of several fields in Nanacamilpa, Tlaxcala, are evaluated for productivity and costs.

**Results:**

Maize grain production is low (1.5 t ha^-1^) and does not cover costs. However, maize stover demands a relatively high price. If it included, a profit is possible (about 110 US $ ha^-1^). We show that non-crop production (weeds for food and forage) potentially has a higher value than the crop. It is only partially used, as there are constraints on animal husbandry, but it diversifies production and plays a role as a back-up system in case of crop failure.

**Conclusion:**

The diversified system described is economically rational under current conditions and labor costs. It is also stable, low-input and ecologically benign, and should be recognized as an important example of integrated agriculture, though some improvements could be investigated.

## Background

Agricultural productivity is defined as the yield of useful product per unit land area [1]. The useful product may have various dimensions, such as biomass, food value, monetary value, energy content, CO^2 ^fixation, and others [2].

Traditional, small-scale, low-input agriculture is generally considered to have low productivity - both in useful biomass and in monetary value as well as net returns. However, some of the highest-yielding agroecosystems have traditional characteristics (mixed cropping, labor-intensive and input-extensive), particularly home gardens [3].

Traditional practices are sometimes prevalent even in regions with good communications and literate farmers with access to information, capital or credit, and external inputs. This is often explained by the conservatism of traditional farmers [4]. But, alternatively, it could suggest that some of these systems may have economic advantages over more high-input forms of crop production.

One such system exists in the maize-growing region of the high valleys and mountains of the center and south of Mexico, with its large urban areas, good communications, relatively good soils and regular rainfall. Modern hybrid varieties and intensive cropping practices have had little success in this area, though they do exist occasionally. The use of chemical fertilizer is common, of herbicides frequent, and of some machinery ubiquitous. However, mixed cropping and a relaxed attitude [5] towards weeds are widespread, as is the use of landrace seed and production mainly for self-consumption. Maize cultivation is often considered to provide a secure basis from which other money-earning activities can be pursued. Since the introduction of large domestic animals by the Spaniards, small-scale farms in the highlands have integrated animal husbandry and cropping, much more than in the more tropical areas of Mexico.

It is well-known that the traditional Mesoamerican maize cropping system is mixed. Maize, beans and squash are the most common combination. However, the trend is toward monoculture, even in relatively traditional systems, often because of government incentives.

One important component of productivity in many traditional [6-12] and not-so-traditional agroecosystems [13] is routinely overlooked in economic analysis: the contribution of wild-growing plants, or non-crop resources [14]. A decade ago, the Hidden Harvest initiative highlighted this omission [15]; however, edible and otherwise useful "weeds" are still not routinely integrated into productivity and value analyses. One reason could be that they are somewhat difficult to quantify, even by farmers themselves. They are certainly not part of the data collected for official statistics, on which most analyses are based.

"Wild" in this context is used in the sense of spontaneous or non-cultivated. It does not mean that humans have had no influence. Many agrestal weeds are highly adapted to their environment. Several examples from Mexico have shown that heavily collected weed populations have a higher allocation to the useful parts, due to selection and promotion *in situ *[12,16-18].

Various studies have emphasized the importance of edible weeds for nutrition, especially of Vitamin A [19-21]. Others have studied quantities consumed by an average rural family, which is usually about 1 - 15 kg fresh mass/week/family during the appropriate season [10,19,22].

The use of weeds for forage in Mexico has drawn some attention [23-25]. Several studies have focused on the quantities of weeds produced [26,27], or on their composition [26,28]. A study in the Valley of Toluca, Central Mexico [22], has shown that the harvesting of the weed vegetation for fodder elevates the economic return of a field by an average of 50% (in some cases much more). These data were based on a quantification of the actual use of these plants. This study was conducted in an area with early-season irrigation and relatively high yields of maize grain.

The next step in this line of inquiry is to document the potential amount and net economic value of the combined useful biomass - cultivated and wild - produced by these semi-traditional maize fields, and in an area dependent on precipitation only. This is the aim of this study.

### The study area

**Figure 1:**
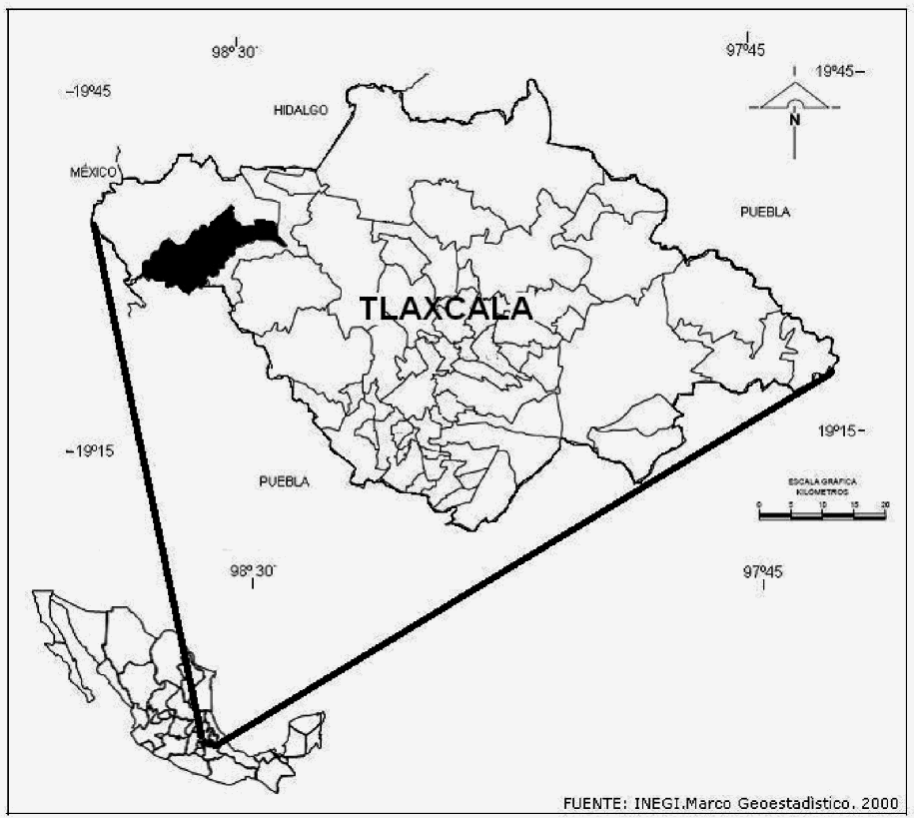
**Location of the study area in Mexico**.

Nanacamilpa de Mariano Arista is situated in the north-west of the small state of Tlaxcala (Fig. 1) at 2,734 m and 19° 27' N, 98° 27 W. The climate is typical of the highland, marginal tropics with a rainy season in summer, a dry season with frosts in winter, and an average annual precipitation of 700 - 1000 mm. The natural vegetation is a pine-oak forest. The main crops of the area are maize, wheat, beans and barley; there are also some *Agave *plantations for the fermented beverage pulque. Temperate fruit trees such as apples, pears, plums, peaches, and the native tejocote (*Crataegus pubescens *(C. Presl) C. Presl) and nopal (*Opuntia ficus-indica *(L.) Mill.) are grown in gardens and along field margins.

Nanacamilpa is a small town of about 15,000 inhabitants, well-linked on a major highway from Mexico City to Tlaxcala and Veracruz. There are schools, some factories, and much commerce, television, telephone and internet access. The surrounding villages have paved roads, schools, public transport and other services. The population is mainly mestizo and Spanish-speaking.

### The cropping system

**Figure 2:**
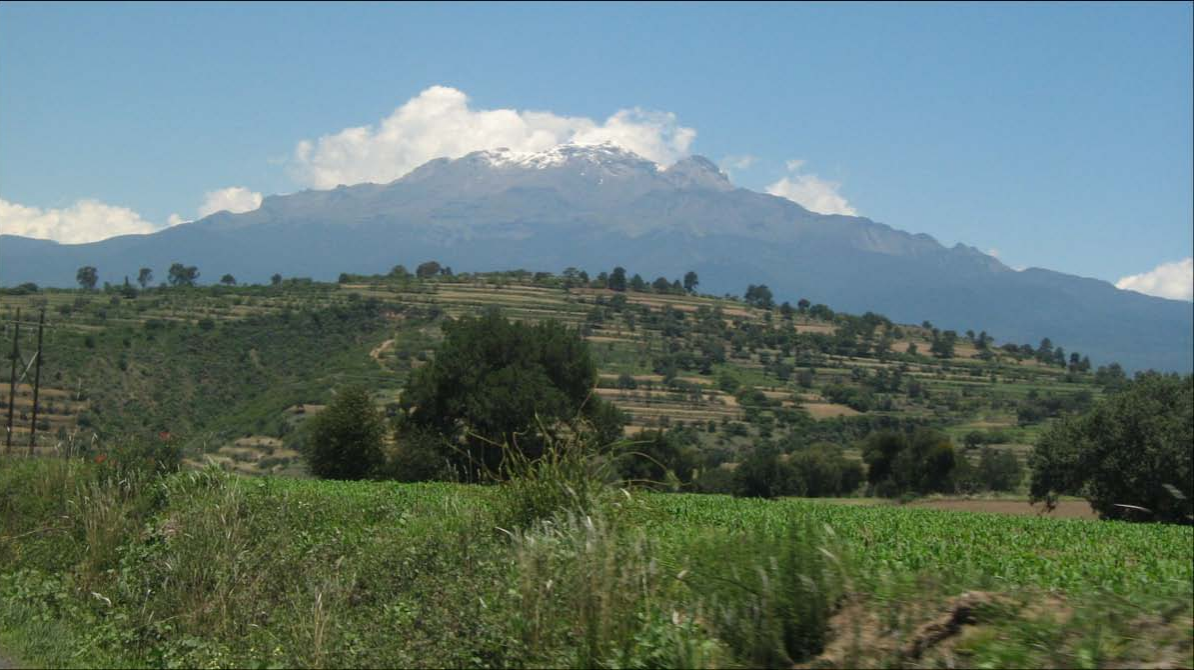
**A typical landscape with terraced maize fields in the study area of Nanacamilpa, Tlaxcala, Mexico, with the Iztaccíhuatl volcano in the background**.

**Figure 3:**
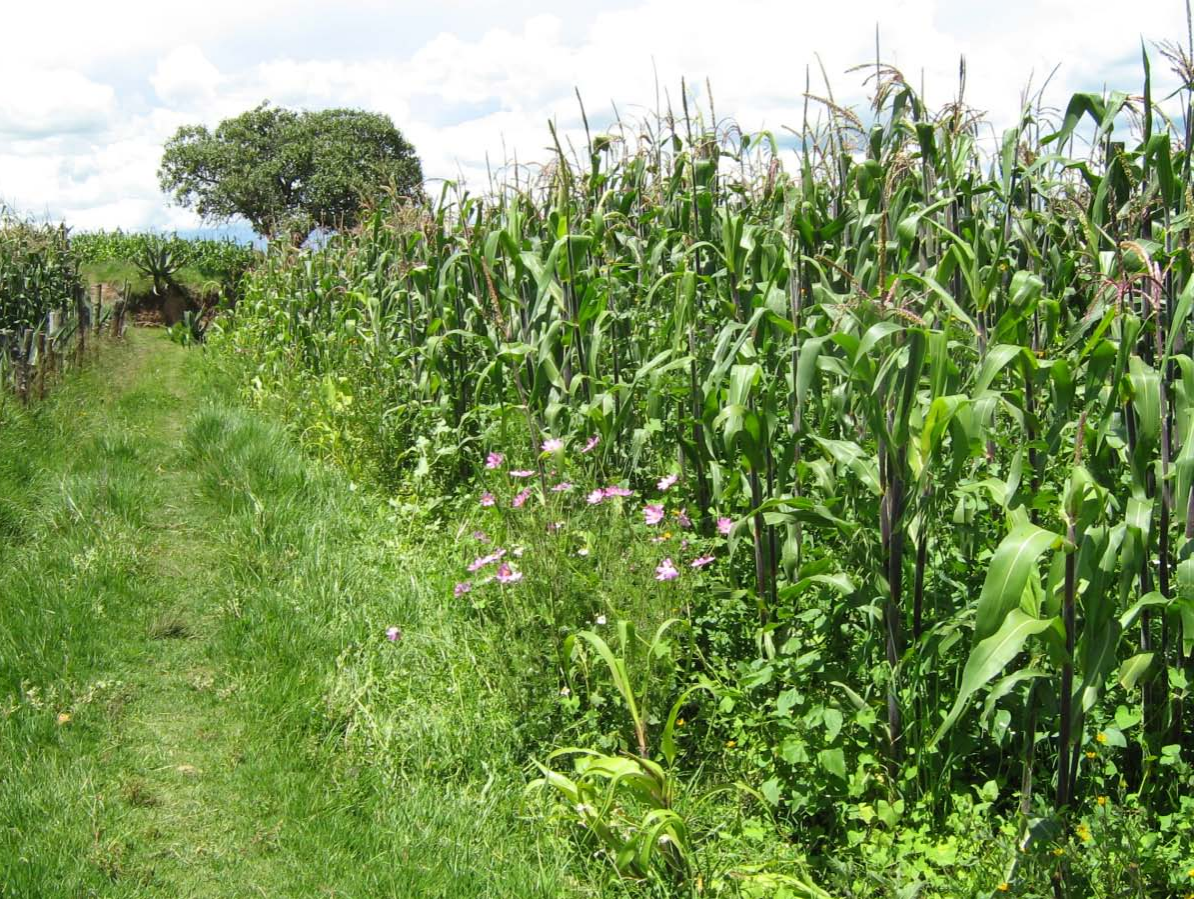
**A maize field in August with weeds allowed to grow between the maize plants**.

The main crop, maize, is cultivated in a terraced landscape (Fig. 2). The soil is plowed between November and February. Shortly before sowing, the field is harrowed and furrows are drawn with a plow, leaving rows 80 cm wide. Traction is either mechanized, by oxen or mules. In March several seeds are hand-sown 25-40 cm apart in the furrows (listering). The germinated seedlings are fertilized a month later with urea or manure. Shortly afterwards, the field is cultivated, to hill the maize and to reduce weed populations. This first cohort of weeds is often cut to be used for food and forage. The field is usually hand-weeded again in the beginning of July; a second fertilization takes place soon afterwards, when the maize starts to form inflorescences. While the maize is kept mostly weed-free during its critical period, from this time on (July), weeds are allowed to develop freely (Fig. 3), and are used mainly as forage. Though some farmers of the region use herbicides, none were used on our study sites. The grain harvest is from November to December. Maize stover is sometimes packed mechanically. No pesticides were used in 2006, though they are known, available and used if there is a larger pest problem. The main risks to agriculture are meteorological - late or early frosts, irregular distribution of rainfall during the rainy season, and hail.

Only one of the surveyed farmers (and none of the study plot owners) used commercial hybrid seeds in zero-cultivation, mainly because of its lower labor requirements (others have tried this system, but were not satisfied). The rest used various landrace seeds. The most common varieties were called criollo (a cream-colored dent) and azul (a blue-black dent); other reported varieties were gavilán (a cream-colored dent with a longer cob) and chalqueño, a cultivar with larger, wider seeds.

**Figure 4:**
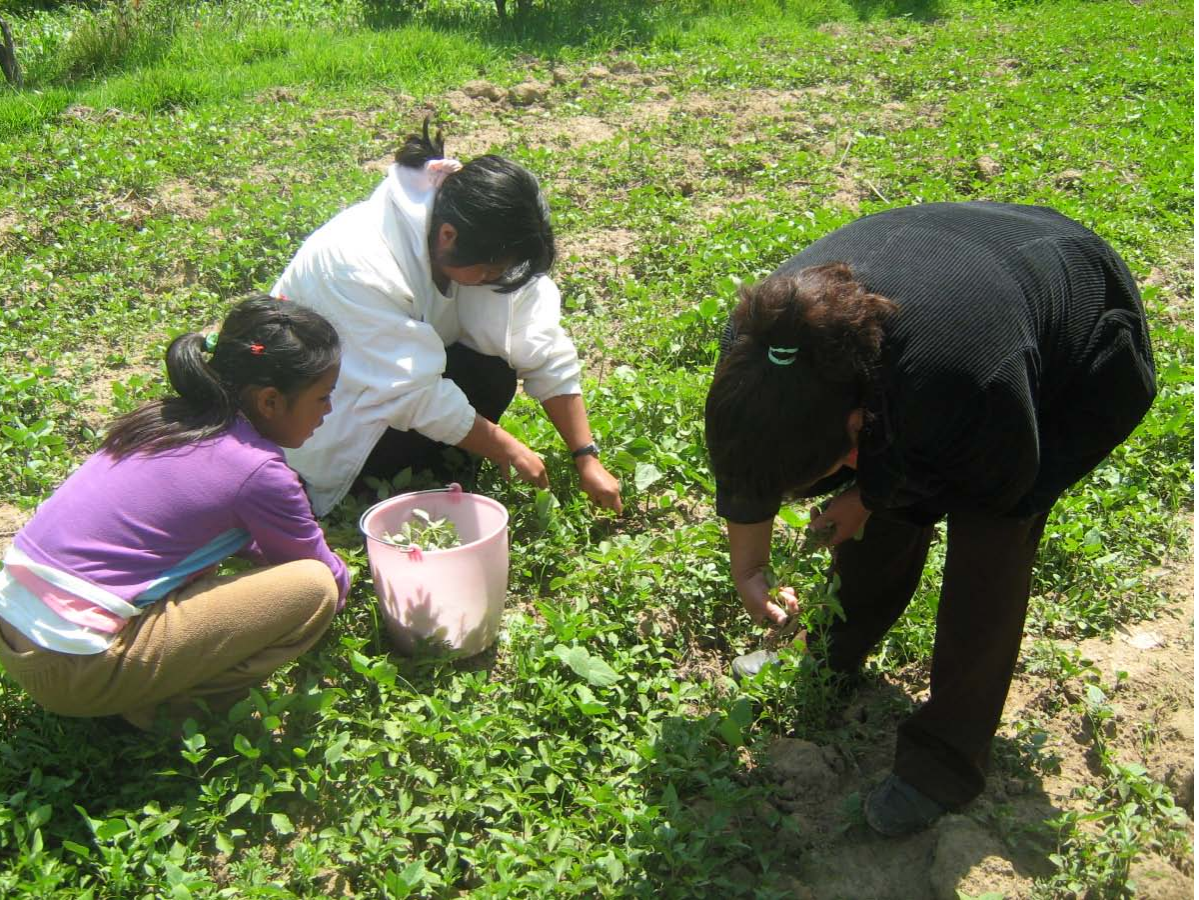
**Harvest of edible wild greens in Nanacamilpa, Tlaxcala**.

**Figure 5:**
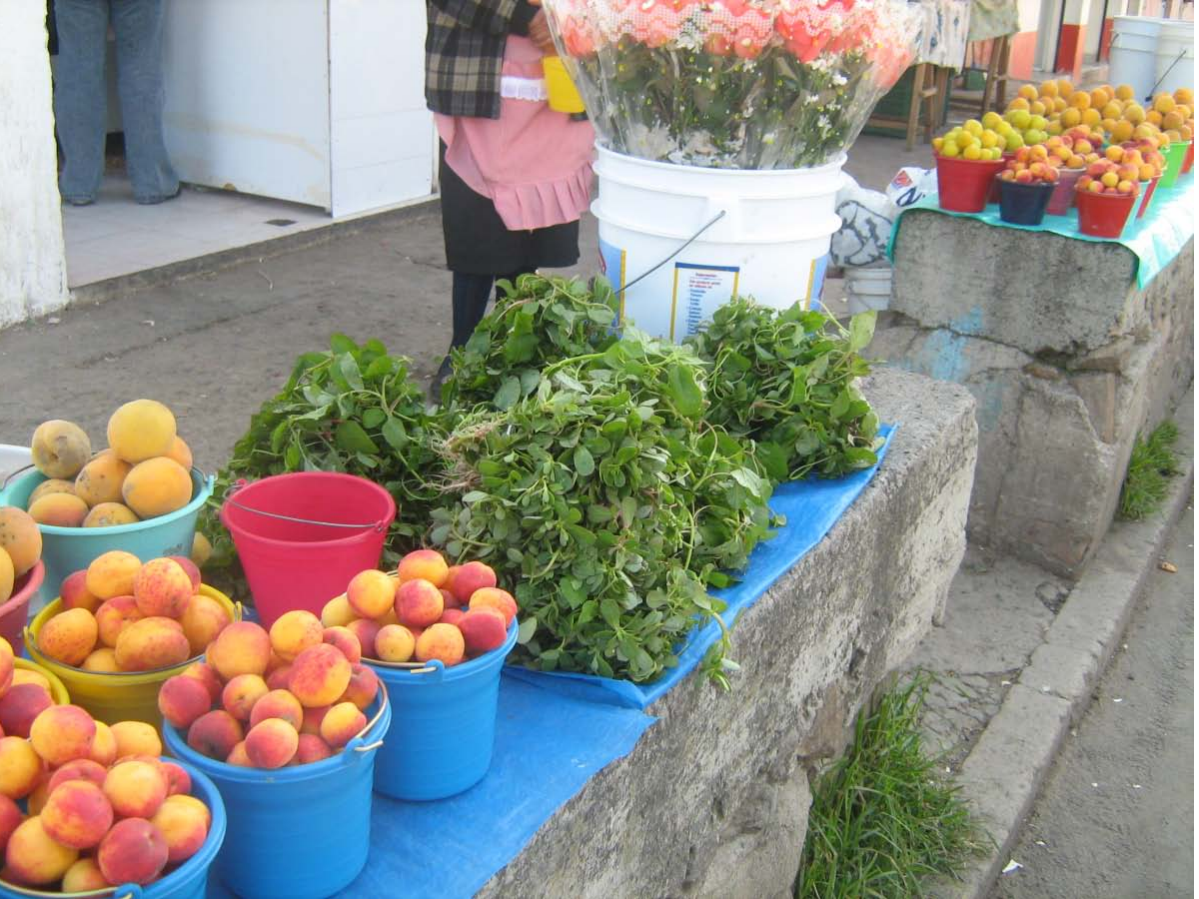
**Sale of edible, wild greens in the Nanacamilpa weekly market (*Portulaca oleracea*, verdolaga, and *Amaranthus hybridus*, quintonil)**.

**Figure 6:**
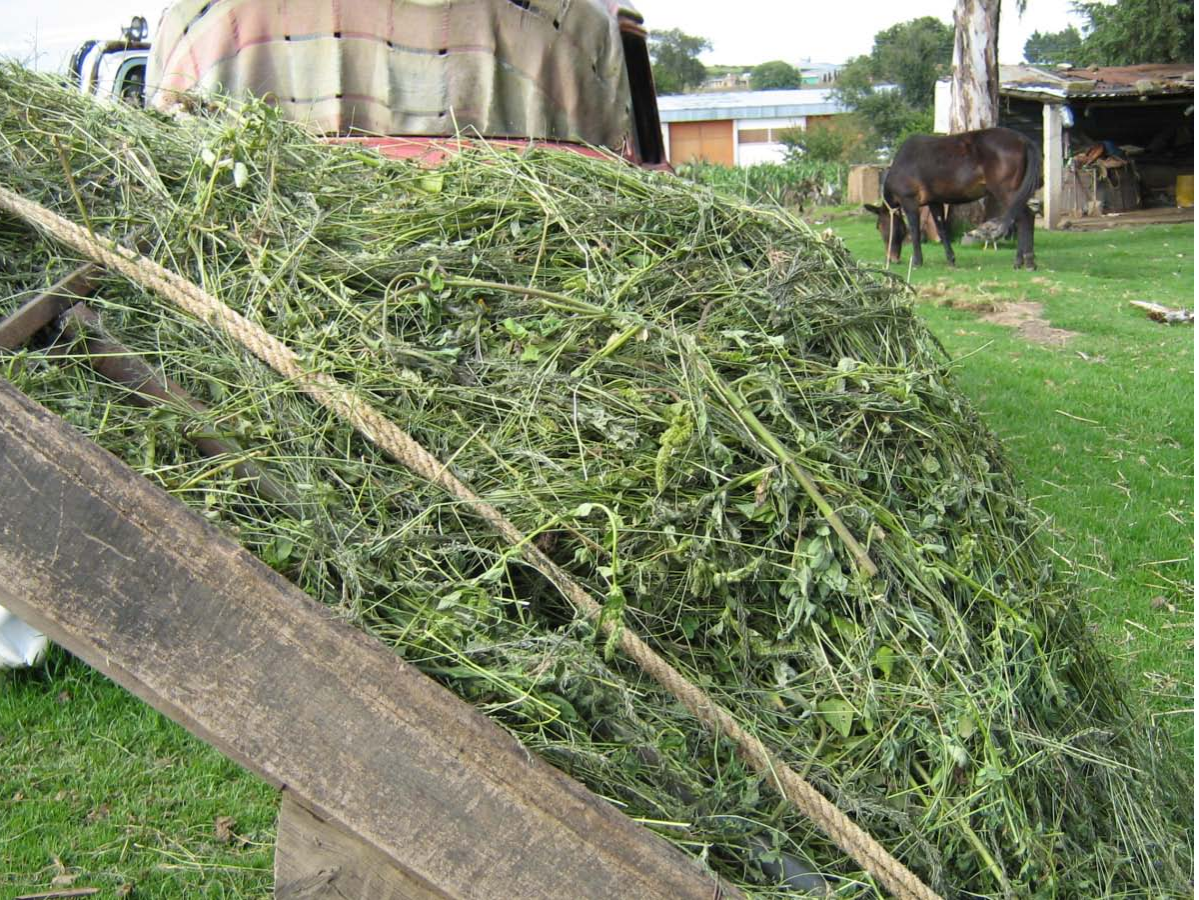
**Harvested weeds mixed with alfalfa for fodder**.

Weeds are harvested in April (mainly for food, Fig. 4, 5) and from July to November (mainly for fodder, Fig. 6).

The study year (2006) was considered a relatively good year for agriculture in the region, with a rainy season that started early, brought sufficient precipitation, and without high winds, late frosts or hail.

## Methods

Written permission was obtained from the local authorities for the field work, and the study protocol was approved by the academic institution. The study was carried out in two parts. First, a systematic botanical collection of maize field weeds and the plants growing on the field margins yielded a species list. The vouchers were deposited at CHAPA and MEXU. Information on plant uses was obtained through interviews of 56 persons in each of three communities (a total of 168) of three age groups and both genders. A field herbarium was used to facilitate the interviews. The 16 adult informants consisted of the owners (man and wife) of four study plots in each of three communities (see below) and four comparable, but non-farming families, selected by a lottery from community archives. Additionally, a group of 20 children (6-13 yr) and 20 teenagers (14-22 yr; 10 boys and 10 girls each) from each of these communities were selected from primary, secondary and high schools of the study area by lottery, and interviewed.

Vendors of quelites (wild potherbs) were interviewed at the weekly market and the permanent market hall to establish commercial species and prices. In each venue three vendors were interviewed twice during the year (Fig. 5). Prices for maize grain and maize stover tended to be standard in the region and were elicited during the interviews and by asking buyers. Prices for non-traded items such as weed forage were established by comparison with farmgate prices of alfalfa and green maize for forage, combined with direct questions on willingness to pay.

The cost of cultivation was considered to be that of contracting out operations (plowing, sowing, cultivation, and so on). Labor input in other activities (for example forage harvest) was established by interviews and by participant observation. The cost of labor was considered to be the regional daily pay for a hired farm hand ("jornal"), which was a relatively high 100 pesos (about 9 US dollars) per day. All costs and prices are in Mexican pesos for 2006; the conversion rate to American dollars (US$) varied between 10.50 and 11 pesos/dollar in that year.

In a second part, production of useful biomass was studied in fields. The study plots were selected from three communities: Nanacamilpa itself, and the villages Miguel Lira y Ortega and San Felipe Hidalgo, located about 2 km of the municipal seat. In each community, 4 fields were selected for geographical dispersal and the willingness of the owners to participate, for a total of 12 study sites. The owners were interviewed on management practices, cost of cultivation, and useful plants.

**Figure 7:**
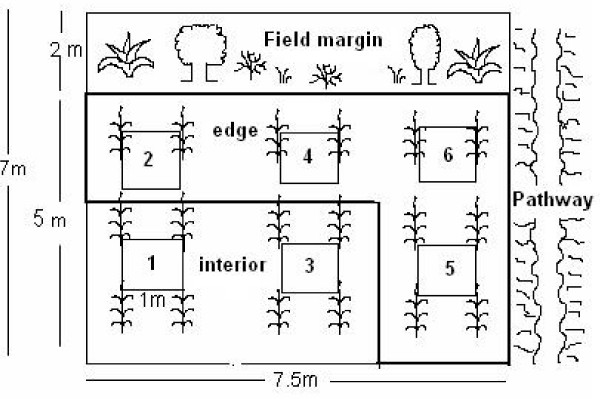
**The position of the sampling plots in the maize field**.

To measure productivity, we marked rectangles of 7.5 × 7 m in a corner of the maize field. Six 1 m^2 ^quadrants were distributed systematically and marked within the rectangle (Fig. 7). Four of these 1 m^2 ^quadrants were considered "edge" and two were considered "interior". We differentiated these because sometimes the vegetation in field borders changes due to better light conditions; also people like to collect wild plants from the margins because they are more accessible. Most fields were not much wider than 15-20 m, so the sampling should be representative.

The biomass of these 6 m^2 ^was harvested twice, in accordance with local custom - in April, before the first cultivation, and in July, when forage harvest commences. The species were registered and separated, the individuals counted and the fresh, above-ground biomass measured. Production of maize grain and stover where determined based on data from four fields.

## Results

### Kinds of useful plants

Of the 109 species encountered in the study plots, almost half (43) were used for forage. Another 39 species had medicinal uses, 18 were considered edible and 10 species had other uses (ornamentals, material for handicrafts, construction material, broom-making and fuel). Only 19 species were not found to have any known use; 33 species had various uses, with the most common combinations being forage-medicinal and forage-food; 4 species had three uses (*Opuntia *spp., *Malva parviflora *L. and *Rumex crispus *L. were used as forage, food and medicinal, and *Muhlenbergia macroura *(Kunth) Hitchc. served as forage, ornamental and construction (roofing) material).

### Commerce with wild plants

The interviews with the market vendors resulted in a list of traded species and their prices (Table 1). The list includes foods, medicinals and condiments. Prices usually rise in the dry season (October to March or April). The quelites (pot herbs) in this list were the most abundant species in the study area, but the vendors usually bought their products at the wholesale market in Puebla.

**Table 1:** Weeds of maize fields commercialized in the permanent and weekly markets of Nanacamilpa de Mariano Arista, Tlaxcala, Mexico (prices in Mexican pesos of 2006 and for fresh weight; 1 US Dollar = 10.50-11.00 pesos in that year).

Species (scientific and common name)	Price Dry season (Oct-March)	Price Rainy season (May-Sept)
*Amaranthus hybridus *(quintonil)	15.00/kg	6.00/kg

*Chenopodium berlandieri *(quelite cenizo)	8.00/kg	5.00/kg

*Calandrinia micrantha *(lengüitas, chivitos)	8.00/kg	5.00/kg

*Malva parviflora *(malva)	8.00/kg	6.00/kg

*Brassica rapa *(nabo)	12.00/kg	5.00/kg

*Gnaphalium *sp. (gordolobo)	32.00-40.00/kg	--------

*Tagetes lucida *(pericón)	---------	24.00-32.00/kg

During the rainy season, vendors had to compete with house-to-house sales of quelites by the farmers; consumers preferred this local produce "because it is sure not to come from irrigated fields" (the products from irrigated fields have a bad reputation because irrigation water is often polluted).

### Production of useful biomass

**Figure 8:**
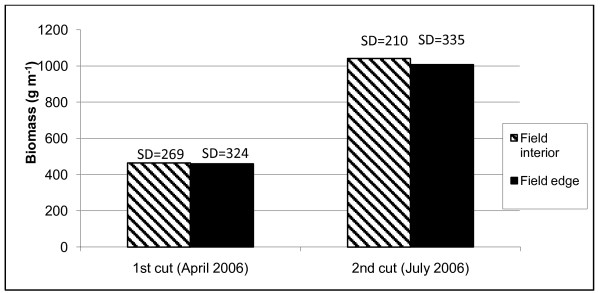
**Comparison of the average fresh biomass produced by weeds in the edge and interior plots (SD = standard deviation)**.

Figure 8 shows the production of fresh biomass of the weed vegetation in the 6 × 1 m^2 ^subplots, divided into edge and interior plots, and for both cuts. The average yield for the first cut was 464 g m^-1 ^for the interior of the field and 459 for the edge and the corresponding yields for the second cut were 1042 g m^-1 ^and 1006 g m^-1 ^respectively. There were no statistically significant differences for differences in plot position (Tukey, p < 0.05), so we pooled the data of all plots for calculating production per hectare.

Useful spontaneous plants contributed 14.8 t ha^-1 ^(fresh weight) on the average, during the whole growing season. Of these, 9.7 t ha^-1 ^were forage species (*Simsia amplexicaulis *(Cav.)Pers.: 6.6 t ha^-1^, *Bidens odorata *Cav. 2.6 t ha^-1^) and the rest were mostly food species (that can also be used as forage). *Amaranthus hybridus *L. produced 3 t and *Chenopodium berlandieri *Moq. 0.7 t per hectare, followed by *Brassica rapa *L. (0.6 t ha^-1^), *Malva parviflora *and *Calandrinia micrantha *Schltdl. (both 0.4 t ha^-1^). Edible plants are usually harvested young, so they will not grow as abundantly as the forage plants that are harvested later. We also observed a curious regularity: there were usually 1-3 edible species in each m^2 ^(Nanacamilpa: 2.1 ± 1.0; Miguel Lira y Ortega: 2.5 ± 0.7; San Felipe 2.0 ± 0.9). Two medicinal/condiment plants, *Rumex crispus *and *Polygonum aviculare *L., produced less than 0.1 t ha^-1^.

Maize production was low: 1.5 t ha^-1 ^in grain and 3.7 t ha^-1 ^(dry weight) in stover (straw, husk and rachis).

### Economic costs and potential benefits of the useful biomass

Table 2 shows the main components of the useful biomass production, maize grain, maize stover, wild vegetables and forage, with their average production, selling price, cost of harvest and net return. From the total, the cultivation cost of 2500 pesos per ha has to be deducted. The average potential net value in 2006 was about 12,000 pesos, that is over 1,000 US $ per ha.

**Table 2:** Economic value of the average biomass production of maize fields in Nanacamilpa in 2006; in Mexican pesos (approximately 10.50 to 11.00 to an American dollar in that year).

Product	Average production	Farm-level selling price	Cost of harvest/commercialization kg^-1^	Gross value	Total cost of harvest/commercialization	Net value
**Forage (fresh weight)**	9.7 ha^-1^	1.00 kg^-1^ (a)	0.40 kg^-1^	9,700 ha^-1^	3,880 ha^-1^	5,820

**Edible plants (quelites; fresh weight)**	5 t ha^-1^	5.00 kg^-1^	4.00 kg^-1^ (b)	25,000 ha^-1^	20,000 ha^-1^	5,000

**Maize stover (dry weight)**	3.7 t ha^-1^	1.00 kg^-1^	8.50/25 kg (packing machine)	3,700 ha^-1^	1,300 ha^-1^	2,400

**Maize grain (dry weight)**	1.5 t ha^-1^	1.30 kg^-1^	20.00 (harvest and degraining of 50 kg)	1,950 ha^-1^	600 ha^-1^	1,350

						14,570.00 - 2,500 cost of cultivation

					**Net economic value of production**	**12,070.00 (ca. 1150 US $)**

a) Amount based on relation with alfalfa (1.30 pesos kg^-1 ^at farm level) and willingness-to-pay data.

b) Whereas the majority of the products are or could be used within the farm, and can be calculated as being worth the farm-gate price of replacement or of a similar product, 5 t of edible herbs clearly exceeds the capacity of consumption of one family. We calculated 3 pesos/kg harvest cost (4 kg harvest/hour, cost of 1 h 12.50 pesos) and 1 peso/kg marketing cost (for transport to wholesaler, or sale within the community). - Edible plants can be used alternatively as forage, where they would have somewhat lower net worth (value is much lower, but costs are also less, because they don't have to be harvested separately by species).

## Discussion

### Kinds of useful plants

The number of plant species found in the maize fields, as well as the number of useful plants and their uses coincides with other studies [12,22]. Also, it is common in central Mexico that only a small proportion of agrestal weeds are not used in some way [22].

### Commerce with wild plants

The interviews show that highly informal channels such as house-to-house sales may be of considerable importance for rural families. This phenomenon was also reported by Vieyra and Vibrans [22], who found that about one third of the harvested quelites were sold within the community. The data also show that rural consumers have preferences as to the quality of the merchandise.

### Production of useful biomass

The production of maize is relatively low, due to the short growing season, and is similar to that reported previously from similar regions. The production of weed biomass is also similar to that reported from other studies; other studies generally report dry weight. The majority of the forage weeds have a dry mass content of about 15-20% [29], so the 14.7 t fresh weight of useful weeds (forage + edible) would have a dry mass of approximately 3 t, almost as much as the maize component. This is similar to the data reported by Díaz [26] with 2.5 t ha^-1 ^in the Valley of Mexico. An experimental trial of cultivation of *Simsia amplexicaulis *as a forage crop in the state of Aguascalientes yielded 3.5 t ha^-1 ^dry mass [30]. Mariaca [27] obtained 1.7 t ha^-1 ^in a different type of ecosystem - the dry tropics of Yucatán - and in an experimental field.

We expected edge plots to have a higher weed production than the interior plots because of higher insolation, but did not find this. The edge effects may be limited to the first meter approximately, and were not detected with our methods.

### Economic costs and potential benefits of the useful biomass

The data show the regional economic importance of maize stover. The stover may command the same price as maize grain (we priced it somewhat lower for our calculations), probably because its price is regulated on a local or regional scale, whereas maize grain competes on the world market. The often-overlooked economic importance of straw production has been noted in other contexts as well, for example in the traditional production of wheat in Afghanistan [11].

The calculations show that the potential benefits from the weed vegetation are higher than those of the main crop. We concur with Maletta [11], who found an active market both for straw and for weeds as fodder, in the economic analysis of wheat cultivation in Afghanistan,:"All these facts underline the necessity of including straw and weeds in the output of the wheat crop. Otherwise the cost of grain would be greatly overstated, and the benefit accruing to farmers greatly undervalued".

The economic valuation shows that these fields have a very high potential net return - about 1000 US $, comparable to some horticultural crops (and much higher than the 100 - 200 US $ ha^-1 ^net return in intensive maize agriculture), but with low external input and high stability of the system. High and Shackleton [14] presented data in the same order of magnitude with an average return of 667 US $ ha^-1^for an African system of home gardens.

Of course, this production is not completely used, because of the seasonal and general constraints on animal husbandry. But it serves as an economical stabilizer, as it can still be used if the main crop fails, for example because of a late frost.

A previous study of a similar region with a much higher maize productivity (because of access to early-season irrigation and therefore a longer growing season) documented that most farmers use some herbicides in order to keep the weed vegetation low to facilitate harvest [22]. However, they left parts unsprayed specifically for the weed harvest. We suggest that this non-crop production is even more important both for direct use and for security in the study region of this work, which is solely dependent on precipitation. Intensification that requires a reduction of the weed population would not easily be profitable, unless there are serious labor constraints. The role of non-crop plant resources in reducing risk of the rural population is often overlooked [14,31].

## Conclusion

This study shows that maize grain - supposedly the main purpose of the cultivation - is actually a minor contributor to total productivity and potential net return in the examined agricultural system, and worth less than the cultivation costs. However, maize straw is worth considerably more, and both together give a modest profit. Wild edible herbs and especially forage make a major contribution, though it is only partially realized.

The spontaneous (weed) vegetation of a traditional agroecosystem can, under certain circumstances, be an economically highly important part of the production, as well as a risk mitigator. It provides a simple explanation why farmers in the highlands of Mexico prefer these less-intensive cropping systems, and why farmers in the tropics are much more likely do adopt intensive systems, as animal husbandry and agriculture are less integrated in these regions.

Moreover, there is room for improvement. As the useful biomass production is concentrated in a few months, it is only partially used because of constraints during the rest of the year. These constraints could be alleviated by conserving the fodder biomass (through dehydration or fermentation), and investigation on these and other low-cost alternatives to improve small-scale meat production.

This work shows that farmer's decisions are economically rational. It emphasizes the need to consider whole systems when evaluating the economic viability of traditional farming methods and for understanding farmer's decisions.

## Competing interests

The authors declare that they have no competing interests.

## Authors' contributions

All authors participated in the planning and design of the work; RGMA carried out the field work; RGMA and HV drafted the first Spanish-language version of the manuscript, which was revised by AMB and FBP; HV wrote the final English-language version. All authors read and approved the final manuscript.
